# Corrigendum to “Simulating Radiotherapy Effect in High-Grade Glioma by Using Diffusive Modeling and Brain Atlases”

**DOI:** 10.1155/2018/2712657

**Published:** 2018-02-28

**Authors:** Alexandros Roniotis, Kostas Marias, Vangelis Sakkalis, Georgios C. Manikis, Michalis Zervakis

**Affiliations:** ^1^Institute of Computer Science, Foundation For Research and Technology-Hellas, 700 13 Heraklion, Crete, Greece; ^2^Department of Electronic & Computer Engineering, Technical University of Crete, 73100 Chania, Greece

In the article titled “Simulating Radiotherapy Effect in High-Grade Glioma by Using Diffusive Modeling and Brain Atlases” [1], there was a typographic error in formula number (11). We would like to thank Borasi and Nahum [2] for identifying this error. However, the correct formula should be as follows.

(11) fc=t∈therapy,ρccm−ccm,t∉therapy.

In addition, we would like to differentiate our work from the strict definition of the Linear Quadratic model. Therefore, we propose changing the name of our model from “Linear Quadratic” model to “Modified Linear Quadratic” model as presented in Wahl et al. [3] cited in the article as reference [43] or we can use the term “*α*-*β* model.” Therefore, the model name should be changed throughout the article.
